# On the Action of Chloroform on the Sensitive Plant (Mimosa Pudica)

**Published:** 1849-06

**Authors:** 

**Affiliations:** Geneva


*On the action of Chloroform on the Sensitive Plant (Mimosa Pudica).* By Professor MARCET, of Geneva.*—When one or two drops of pure chloroform are placed on the top of the common petiole

* Read before the *Société de Physique et d'Histoire Naturelle*, October 19, 1848, and communicated by the author.

of a leaf of the sensitive plant, this petiole is seen almost immediately to droop, and an instant after the folioles close successively, pair by pair, beginning with those which are situated at the extremity of each branch.* At the end of one or two minutes, sometimes more, according as the plant is more or less sensitive, most of the leaves next to the chloroformed leaf, and situated beneath it on the same stalk, droop one after another, and their folioles contract, although generally in a less complete manner than those of the leaf placed in immediate contact with the chloroform. After a rather long time, varying according to the vigour of the plant, the leaves open again by degrees; but on trying to irritate them by the touch, it is seen that they have become nearly insensible to this kind of excitement, and no longer close as before. They thus remain as if torpid for some time, and generally do not recover their primitive sensitiveness till after some hours. If, however, when they are in this state of apparent torpidity, they are subjected again to the action of the chloroform, they close as they did the first time. It is not until they have been chloroformed several times, they lose all kind of sensitiveness, at least until the next day; sometimes they even fade completely at the end of too frequent repetitions of the experiment. In all cases the effects observed are more marked in proportion to the purity of the chloroform employed, and the degree of sensitiveness in the plant.

An analogous phenomenon is produced if, instead of placing the drop of chloroform on the base of the petiole, it is laid on the folioles situated at the extremity of a branch. The folioles of this branch immediately begin to close pair by pair, the common petiole droops, lastly the folioles of the other branches close in turn. At the end of two or three minutes, the nearest opposite leaf, and if the plant is vigorous, most of the leaves situated below on the same stalk, follow their example. When, after some time, the leaves open again, the same want of sensitiveness is manifested as in the preceding case.

A singular feature in this phenomenon is the manner in which the action of the chloroform is propagated from one branch to another, then from one leaf to another, even when the liquid disappears almost as soon as it is deposited. This action, as we have just seen, appears to be communicated from the leaf to the stalk, following in the latter a descending direction; generally the leaves situated beneath the chloroformed leaf are not at all affected. De Candolle, in making an analogous experiment on a sensitive plant with a drop of nitric, or sulphuric acid, remarked, on the contrary, that it was the leaves above the leaf touched which closed, without those situated beneath participating in this motion.† The observation of our learned countryman, is quite naturally explained by attributing to the ascending sap the transport of the corrosive poison, a transport which, in this case, would take place in the direction from below upwards. But how to account for the apparent

* I previously convinced myself by experiment, that a drop of water, placed delicately on a leaf of the sensitive plant, caused no movement.

† De Candolle, Physiologie Végétable, vol. ii. p. 866.

transmission of the effects of the chloroform in the contrary direction, from above downwards? Might the descending sap more peculiarly have the effect of transmitting the narcotic effects of this singular compound from one part of the sensitive plant to the other; or might there exist in this plant some special organ susceptible of being affected by certain vegetable poisons in a manner analogous to the nervous system of animals? Notwithstanding the interesting investigations of Dutrochet and other physiologists, there still prevails too much obscurity on this subject to hazard an opinion. But in any case the fact is singular, and appears to me to merit the attention of persons accustomed to engage in questions of this nature.

Experiments of the same kind, made on the contractility of the sensitive plant with rectified ether, have furnished me with results nearly similar to the preceding; with this difference, however, that whilst one drop of chloroform placed on the common petiole of a leaf situated at the extremity of a branch of a sensitive plant, suffices to cause most of the other leaves, situated beneath, on the same branch to close; ether, in general, produced effect only on the leaf itself with which it is put in contact. The next leaves have generally appeared to me not affected. I must say, however, that my experiments with ether having been made after the others, and at a time of year when the sensitiveness of the plant already began to diminish, it is possible that the intensity of the effects produced may have thereby been affected.

